# Mechanistic Insights into the Anticancer Potential of Methoxyflavones Analogs: A Review

**DOI:** 10.3390/molecules30020346

**Published:** 2025-01-16

**Authors:** Mohammad Aidiel, Maisarah Abdul Mutalib, Rajesh Ramasamy, Nik Nasihah Nik Ramli, Shirley Gee Hoon Tang, Siti Hajar Adam

**Affiliations:** 1 School of Graduate Studies, Management & Science University, University Drive, Off Persiaran Olahraga, Section 13, Shah Alam 40100, Malaysia; aidiel980429@gmail.com (M.A.); niknasihah_nikramli@msu.edu.my (N.N.N.R.); 2 Department of Pathology, Faculty of Medicine and Health Science, Universiti Putra Malaysia, Serdang 43400, Malaysia; rajesh@upm.edu.my; 3 Center for Toxicology and Health Risk Studies (CORE), Faculty of Health Sciences, Universiti Kebangsaan Malaysia, Jalan Raja Muda Abdul Aziz, Kuala Lumpur 50300, Malaysia; shirleytgh@ukm.edu.my; 4 Preclinical Department, Faculty of Medicine & Defence Health, Universiti Pertahanan Nasional Malaysia, Kuala Lumpur 57000, Malaysia; siti.hajar@upnm.edu.my

**Keywords:** medicinal plants, methoxyflavones, physicochemical properties, structural-activity relationship, anticancer mechanisms

## Abstract

2-phenylchromen-4-one, commonly known as flavone, plays multifaceted roles in biological response that can be abundantly present in natural sources. The methoxy group in naturally occurring flavones promotes cytotoxic activity in various cancer cell lines by targeting protein markers, in facilitating ligand–protein binding mechanisms and activating cascading downstream signaling pathways leading to cell death. However, the lipophilic nature of these analogs is a key concern as it impacts drug membrane transfer. While lipophilicity is crucial for drug efficacy, the excessive lipophilic effects in flavonoids can reduce water solubility and hinder drug transport to target sites. Recent in vitro studies suggest that the incorporation of polar hydroxyl groups which can form hydrogen bonds and stabilize free radicals may help overcome the challenges associated with methoxy groups while maintaining their essential lipophilic properties. Naturally coexisting with methoxyflavones, this review explores the synergistic role of hydroxy and methoxy moieties through hydrogen bonding capacity in maximizing cytotoxicity against cancer cell lines. The physicochemical analysis revealed the potential intramolecular interaction and favorable electron delocalization region between both moieties to improve cytotoxicity levels. Together, the analysis provides a useful strategy for the structure–activity relationship (SAR) of flavonoid analogs in distinct protein markers, suggesting optimal functional group positioning to achieve balanced lipophilicity, effective hydrogen bonding, and simultaneously minimized steric hindrance in targeting specific cancer cell types.

## 1. Introduction

**Figure 1:**
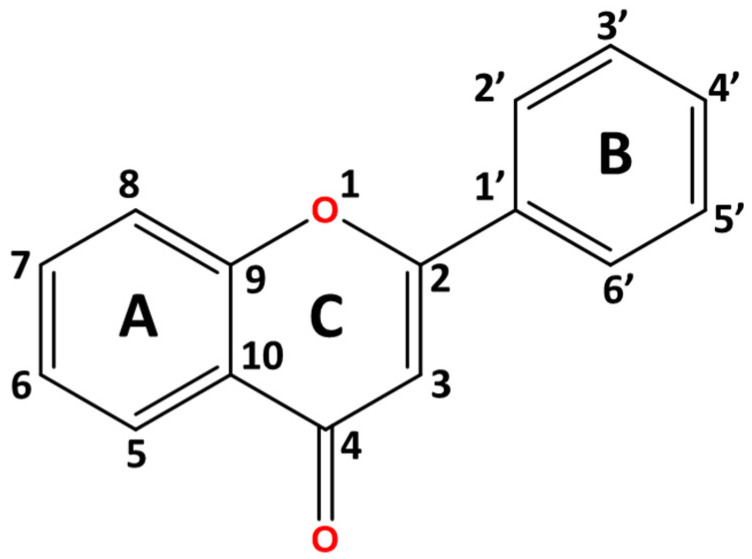
Skeleton structure of flavone analogs.

Flavones, a prominent subclass within the flavonoid family, exhibit a multi-target pharmacological profile owing to their intricate structural system. This structure features a chromone core (1-benzopyran-4-one) with a hydrophobic phenyl ring attached to the pyran ring (Figure 1) [1]. The unique pharmacological properties of natural flavonoid, particularly flavones, contribute to its diverse bioactivities in both traditional therapies and modern medicine [2,3,4,5]. The physicochemical properties of these flavones are largely attributed to two major groups that coexist, predominantly the natural methoxy and hydroxy substituents [6,7]. Both groups exhibit distinct characteristics and work synergistically in an electron donor–acceptor mechanism, influencing electron density distribution depending on their position in rings A, B, and C of the flavone structure [8,9,10]. As a result, the flavone pharmacophores significantly influence the bioavailability of flavones, one of the primary focuses in discovering potential therapeutic drugs [11]. Drug discovery originating from medicinal plants has fruitfully developed new potent therapeutics, such as morphine, an alkaloid derived from *Papaver somniferum* (opium poppy) [12]. Similarly, the discovery of taxol, a terpenoid compound from *Taxus brevifolia* (pacific yew tree) led to the commercialization of paclitaxel as an antitumor drug [13]. Despite the reported biological efficacy of flavones, a comprehensive systematic review authored by Xu et al. [14] highlighted significant challenges in developing anti-tumor drugs based on flavones aglycones. These challenges are often due to physical and structural limitations that affect the bioavailability. Thus, efforts to delve into the chemistry of highly lipophilic flavones were enormous, particularly focusing on the hydrophobic region such as the unsaturated double bond between C2 and C3 on ring C and phenyl ring B.

The distinct physicochemical attributes of methoxy and hydroxy moieties add a prospect to the synergistic relationship between both functional groups in enhancing biological responses. Therefore, the in-depth analysis of the physicochemical properties, including lipophilic effects, intramolecular interactions, and hydrogen bonding capacity will be useful in the structure–activity relationship (SAR) investigations of methoxyflavones’ analogs to determine its target of interest and eventually contribute to its distinct biological activities [15,16,17]. An optimal lipophilic capacity of a lead compound is part of the major limitation of flavones in anticancer drug discovery [18,19]. The partition coefficient, logP, and distribution coefficient, logD, is one of the parameters to predict hydrophobicity and lipophilicity and has been validated on various flavone scaffolds [20]. The lipid–water partitioning of flavones is crucial given its influence on the uptake and delivery of flavones to the target sites. In plant species, methoxy moieties within the flavones scaffold strengthen the hydrophobic interaction through methylation, possessing hydrogen bonding acceptor (HBA) capabilities and initiating a stabilized ligand–protein binding energy [17,21]. Despite its greater binding and interaction with the active sites, the methoxylated group is a metabolic liability to a compound that leads to biotransformation through the *O*-demethylation metabolic pathway [22,23]. Although not reported for every naturally isolated and synthesized methoxyflavones scaffold, methoxylation could alter bioavailability, solubility, and pharmacology in flavones [24,25,26]. Nevertheless, the presence of the hydroxy group within the methoxyflavones scaffolds could offset the drawbacks by altering the extreme lipophilicity of flavones through intramolecular interaction and a greater electron delocalization region in stabilizing the free radicals and establishing a strong interaction with the target binding sites such as hydrogen bond donor (HBD) [27,28]. However, the incompatible position of the highly polar hydroxyl group within the flavones’ pharmacophores may activate unfavorable thermodynamic properties and desolvation penalties which pose significant challenges in medicinal chemistry [29,30].

The subtle repositioning of hydroxy and methoxy groups on the flavone ring greatly affects the biological activity and the application of the flavones’ analogs either in the hydrophobic or polar aqueous region [31]. The coexistence of a hydroxy functional group may expand the polar region through hydrogen bonding interactions, though this effect depends on its position [32]. The hydroxy group in flavonoids including flavones has been extensively researched for its ability to scavenge free radicals, diminish ROS levels, and contribute to its potency as antioxidant, anticancer, and anti-inflammatory agents [33,34,35,36,37]. Hydrogen bonding of the hydroxyl group is notably robust and crucial for acting as a hydrogen bond donor. Nevertheless, an enormous polar region might accelerate drug hydrolysis and impair lipid membrane permeation [20]. Despite challenges related to metabolic stability and lipophilicity, methoxy moieties in flavones analogs have shown selectively potent anti-inflammatory effects, enhanced by the C4′-methoxyl group [38] and an increased cytotoxic effect from C7-methoxy substitution [16]. In addition, the existence of both methoxy and hydroxy functional groups in adjacence and its impact on the stereospecificity of flavones merits further exploration [16]. These interactions could promote the formation of an intramolecular interaction between the two atoms, serving as a double-edged sword by improving the stability of the parent structure and potentially blocking polar interaction in favor of a lipophilic environment through the shielding effect of intramolecular hydrogen bonding (IHB) [39,40]. The IHB between a hydroxyl group adjacent to the carbonyl group in various aromatic and polyphenolic compounds has been reported in computational studies [41,42,43]. For instance, the C5-OH substituent within the flavone skeleton interacts with the C4 carbonyl group through intramolecular hydrogen bonding, with multiple studies showing that the associated increase in lipophilic capacity enhances cytotoxic effects on cancer cell lines more than C5-OCH_3_. The elevation in lipophilicity was observed in an earlier study by Whaley et al. [44], when the measured and theoretical logP values were highest on the C5-OH position, followed by C7-OH and C4′-OH. Based on the distribution coefficient, the logD value of C5-OH was higher than the C5-OCH_3_, which indicates the strength of IHB in elevating the lipophilic capacity [44]. Additionally, the methoxy group acting as a hydrogen bond acceptor (HBA), may also form IHB in optimally positioned flavones analogs, particularly when ortho to the hydroxy moieties. The degree of HBD potential of C5-OH investigated through ^1^H-NMR was the weakest, with an Abraham summation solute hydrogen bonding acidity (A) value of 0.02. Meanwhile, the presence of a hydroxy group on ring B (C2′, C3′, C4′) demonstrated the highest A value, indicating strong hydrophilicity capacity [45]. The A value factors in the possible self-dissociation of the hydroxy group in the logP and logD investigation that contributed to the distinct theoretical and experimental values. Through proton NMR, the presence of C3′-OCH_3_ lowers the hydrogen bonding acidity of C4′-OH by 20%, suggesting a stronger potential for stable IHB formation between the OH and OCH_3_ groups, thereby increasing lipophilic character [45,46]. Nevertheless, stable IHB formation can restrict the hydrogen donor capacity of C4′-OH and reduce its affinity for target proteins, unless compensated for by the external interaction with protein markers. In flavones, the intramolecular hydrogen bonding with the adjacent atoms predominantly occurs in rings A and B. Given their distinct roles in polar–hydrophobic interactions, a balanced relationship between these moieties is crucial for maximizing the pharmacological potential of flavones, particularly in anticancer applications. In this updated review, the latest data were gathered from multiple databases including SCOPUS, WOS, PUBMED, and Google Scholar. We believe the in-depth analysis would provide a valuable insight to target and designing future hydroxy and methoxyflavones analogs.

## 2. SAR and Mechanism of Anticancer Activity of Methoxyflavones Derivatives

### 2.1. Breast Cancer

**Figure 2:**
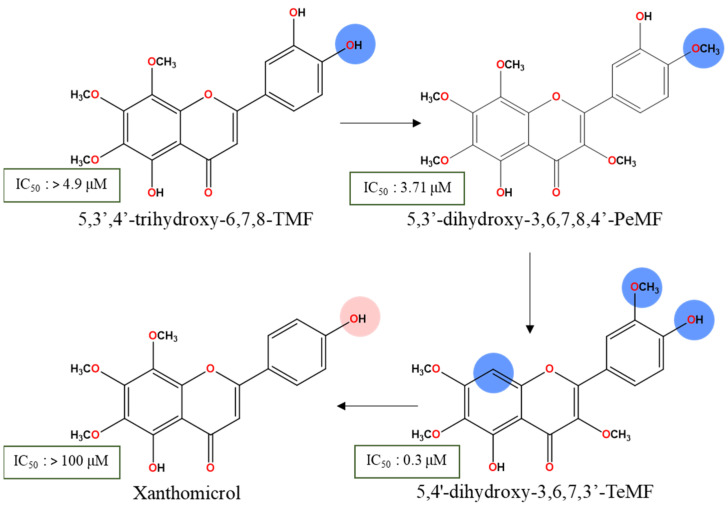
The influence of the position of both functional groups in ring B on the IC_50_ value in MCF-7 in 72 h treatment duration [47,48,49]. The presence of both methoxy and hydroxy moieties on neighboring positions (C3′ and C4′) is a crucial factor for a stronger IC_50_ of 5-OH methoxyflavones analogs. The C6,7,8-OCH_3_ plays a crucial role in stabilizing the lipophilic capacity of ring A flavones. Given the presence of C6 and C7-OCH_3_, the reduction of single C8-OCH_3_ will not significantly affect the IC_50_ of these scaffolds. For xanthomicrol, the absence of methoxy adjacent to the C4′-OH was a single factor in the expected weak cytotoxicity profile.

Breast cancer cell lines were among the most extensively studied on the cytotoxic capacity of methoxyflavones analogs. Sideritoflavone, 5,3′,4′-trihydroxy-6,7,8-TMF, demonstrated a strong cytotoxic effect on MCF-7 cell lines after a 72 h treatment, with an IC_50_ of 4.9 μM [47]. By replacing C4′-OH with the methoxy group (Figure 2), the compound 5,3′-dihydroxy-3,6,7,8,4′-PeMF, isolated from *Glycosmis ovoidea*, demonstrated stronger IC_50_ values of 3.71 μM in a similar treatment duration [48]. Signs of apoptosis such as cell membrane blebbing, shrinkage, and fragmentation were observed. A loss of mitochondrial membrane potential at concentrations of 2 and 20 μM was noted, indicating that intrinsic apoptosis had occurred [49]. 

**Figure 3:**
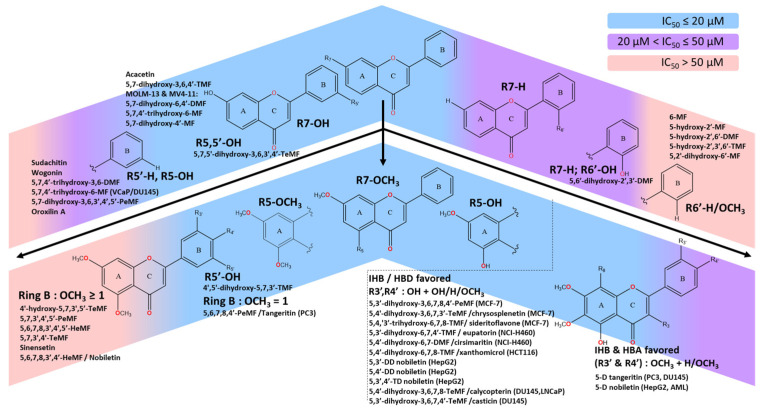
Schematic diagram on anticancer activity of methoxyflavones scaffold. The C7 position plays a key role in the cytotoxic activity on cancer cell lines.

**Figure 4:**
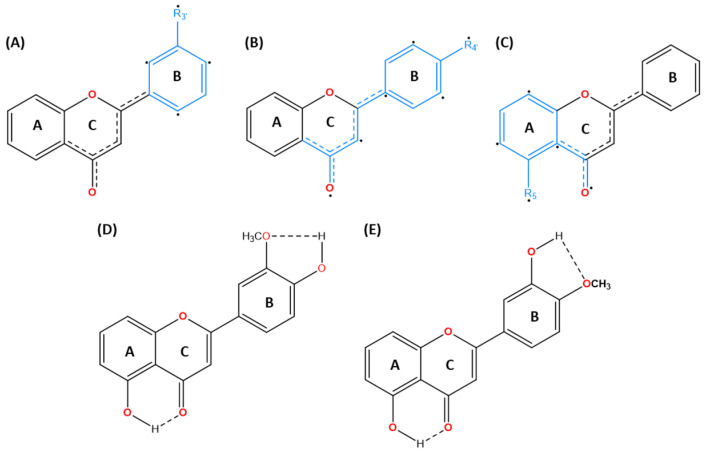
Lewis’s resonance structures illustrating electron delocalization in (**A**) C3′, (**B**) C4′, and (**C**) C5 free radicals. Ring AC and BC are not conjugated. According to a 2013 study, a strong IHB between C5-OH and the C4 oxygen atom of the carbonyl group facilitates the formation of IHBs involving (**D**) C4′-OH and (**E**) C3′-OH with an adjacent methoxy groups.

By swapping the hydroxy and methoxy groups’ positions from C4′ to C3′, and removing C8-OCH_3_, chrysosplenetin (5,4′-dihydroxy-3,6,7,3′-TeMF) was isolated and effectively induced cancer cell death on MCF-7 in a 72 h treatment with a stronger IC_50_ value of 0.3 μM [50]. Another methoxyflavones, xanthomicrol, 5,4′-dihydroxy-6,7,8-TMF, was inactive with an IC_50_ exceeding 100 µM [47]. Based on the schematic diagram (Figure 3), the 3,6,7-TMF scaffold is the backbone that preserves the lipophilic character, acts as a potential hydrogen bond acceptor, and facilitates the hydrophobic interaction with the target proteins. The delocalization region of electron density from C5-OH free radicals (Figure 4) enables a highly stabilized intramolecular hydrogen bond (IHB) with the adjacent carbonyl group. This IHB between C5 and C4 substituents elevates the lipophilic capacity of the flavones’ analogs enhancing drug–lipid transfer and cytotoxic activity [45,51]. Interestingly, the interchanging effect of neighboring moieties on ring B significantly influences the cytotoxicity in MCF-7 reducing the IC_50_ from 4.91 to 0.3 µM, which highlights the favorable positioning of the mono C4′ hydroxy group over C3′ and dihydroxylated group on ring B. Regardless, both positions are within the ideal region for maximizing the roles of the hydroxy group to intensify the anticancer effect of methoxyflavones analogs. Similarly, compared to C5-OH, C4′-OH free radicals generate a stronger resonance effect than the C3′ attributed to the extended pi-conjugation from the C2–C3 unsaturated bond up to carbonyl ring C (Figure 4) [27]. The existence of stabilized hydrogen bonding between C5-OH and the neighboring carbonyl group, along with the resonance effect from C4′ free radicals, assist in their interaction with C3′-OCH_3_, preserving the conformation and shielding the polar surface on ring B against a specific target [52,53,54,55]. As a result, the absence of a neighboring methoxy group next to C4′-OH (xanthomicrol) leads to poor bioactivity. Thus, greater reduction potential from the resonance effect paves the way for strong hydrogen bonding with protein markers. The removal of C8-OCH_3_ unexpectedly preserved the biological effect of the scaffold. Due to the limited data available to predict the roles of C8-OCH_3_, it is hypothesized that for specific cell lines, the methoxylation of at least three positions within the AC-ring system from C5-OH methoxyflavones would be accommodative in balancing its physicochemical properties. C8-OCH_3_ may not necessarily contribute to the cytotoxic effect against MCF-7, even with the presence of heavily substituted C3,6,7-OCH_3_ in the methoxyflavones scaffold.

Focusing on triple-negative breast cancer cell lines, a similar compound 5,3′-dihydroxy-3,6,7,8,4′-PeMF was compared with nobiletin, 5,6,7,8,3′,4′-HeMF, for cytotoxicity activity against MDA-MB-231 cell lines. As anticipated, compound 5,3′-dihydroxy-PeMF significantly reduced cell viability with an IC_50_ of 21.27 μM, compared to nobiletin which showed ~25% reduction at 10 μM, with no IC_50_ reported after a 72 h treatment. A marginal increase in IC_50_ in MDA-MB-231 cells compared to MCF-7 may be attributed to their highly invasive and metastatic nature. Several factors contribute to nobiletin inactivity including, though not restricted to, (i) extreme lipophilic effect of ring B, (ii) exacerbated steric hindrance, and (iii) absence of IHBs that stabilize the pharmacophore, leading to compound degradation. Despite the similarities in functional groups between the two compounds except for the C3 hydroxy group, the role of hydroxy groups in the antitumor activity of methoxyflavones was noteworthy. In an in silico investigation, the hydroxy group was a major contributor to the formation of hydrogen bonding with protein markers associated with the cancer cell death mechanism. For instance, the formation of hydrogen bonding arising from the C5-OH will be influenced by the strength of IHB with the C4 carbonyl group [56]. Nevertheless, greater resonance strength of free radicals on C3′ and C4′ ring B by the hydroxy group established a strong hydrogen bonding interaction with various protein markers in cancer pathogenesis, leading to lower IC_50_ values [56,57,58,59]. The direct apple-to-apple comparison may be premature to definitively claim that hydroxy flavones are the key markers in flavone analogs’ effectiveness. As opposed to the methoxy groups, reports indicate that hydroxyflavones analogs’ cytotoxicity against MDA-MB-231 cell lines was significantly lower with IC_50_ values exceeding 200 μM for treatments ranging from 24 to 72 h, compared to the hexamethoxyflavones compound, nobiletin(>30 µM) [60]. The reduced effectiveness is attributed to the extensive polar surface of polyhydroxylated flavones [61,62,63]. By incorporating both distinct moieties on ring B flavones, compound 5,3′-dihydroxy-3,6,7,8,4′-PeMF could achieve a greater cytotoxic effect on more invasive MDA-MB-231 cell lines with an IC_50_ value of 21.27 µM in a 72 h treatment. Thus, strategically optimizing the placement of hydroxy moieties within the methoxyflavones pharmacophore could balance the extreme lipophilic and polar region features, enhancing their synergistic interaction.

**Figure 5:**
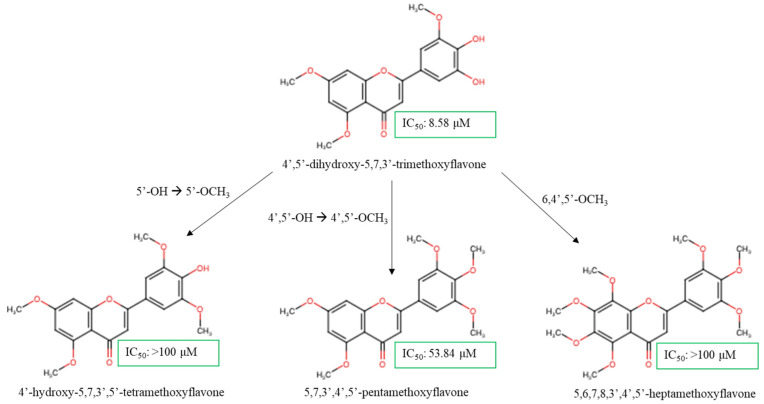
Ring B dihydroxylated moieties highlight the impact of C5′ scaffold composition to the cytotoxic effect on HCC1954 breast cancer cell lines [64]. The presence of C5-OCH_3_ shows enhanced cytotoxicity similar to the C5-OH methoxyflavones scaffolds, influenced by C5′-OH. As summarized in Figure 3, an excessive methoxylated effect on ring B yielded a negative IC_50_ result on cancer cell lines.

The polymethoxyflavones were screened against human epidermal growth factor receptor 2 positive (Her-2+) HCC1954 breast cancer cell lines in vitro [64]. Compound 4′,5′-dihydroxy-5,7,3′-TMF exhibited strong cytotoxicity against these cancer cells with an IC_50_ value of 8.58 µM (Figure 5). The cytotoxicity of the treatment was marginally weaker compared to the previous study that was discussed due to the more invasive and metastatic nature of HCC1954 relative to MCF-7 [65]. Surprisingly, dehydroxylated moieties on ring B increased cytotoxicity activity. The formation of IHB between C4′ and C5′ hydroxy groups was found to be active, greatly stabilized the scaffold, and preserved the lipophilic properties of the flavones [27]. However, modifying the skeleton by substituting C5′-hydroxy to C5′-methoxy group (4′-hydroxy-5,7,3′,5′-TeMF) significantly weakened the cytotoxic effect, resulting in an IC_50_ exceeding 100 µM. The comparison between both compounds highlights several challenges including the bulkiness of the C5′-OCH_3_ group that hindered IHB formation with the carbonyl group and the presence of multiple methoxylated substitutions on ring B (C3′ and C5′) which were unfavorable. Without C5-OH, the C4′-OH in compound 4′-hydroxy-5,7,3′,5′-TeMF exhibited unexpectedly poor cytotoxicity, triggered by an increased potential for a resonance effect arising from C4′-OH free radicals to the carbonyl group, as both AC- and BC-ring electron delocalizations were not in the conjugated system [66,67]. The C4′-hydroxy regions of flavone pharmacophores are known for having the weakest hydrogen bonding energy in ring B and are the most active radicals that readily distribute electron density with a greater active region rise from ring B up to the carbonyl group of ring C from resonance and conjugation effects [68]. Thus, an excessive polar surface significantly weakens the bioactivity of the scaffold. Fully methoxylated flavones, 5,7,3′,4′,5′-PeMF, demonstrated a better IC_50_ at 53.84 µM, although the range was considered weak as the IC_50_ exceeded the threshold of 50 µM. With a limited polar surface, the massive lipophilic capacity from C3′,4′, and 5′-trimethoxy substitution may enhance membrane transfer but will restrict strong hydrogen bonding interaction with the protein target markers. As a result, the addition of two more methoxy groups on C-6 and C-8 (5,6,7,8,3′,4′,5′-heptamethoxyflavone) resulted in weak cytotoxic effects on the cell lines with an IC_50_ > 100 µM. We hypothesize that high methoxy substituents on ring A of the flavone structure, with the absence of a polar region, worsens the active region of methoxyflavones, thus suppressing the cytotoxic effect on HCC1954 breast cancer cell lines. Nevertheless, the SAR analysis revealed that C5′-OH contributes to enhancing the cell death effect on HCC1954 cell lines. An in-depth investigation into the incorporation of C5′-OH with the methoxyflavones scaffold may be required as current data are limited to validate the strength of the C5′ position. 

### 2.2. Prostate Cancer

The anticancer effects of methoxylated flavones have been extensively studied against various prostate cancer cell lines in vitro, including PC3, VCaP, LNCaP, and DU145. Recent research involving six methoxyflavones across five studies revealed that all flavones derivatives bearing C6-OCH_3_ incorporated into their skeleton structure (Figure 3). The variations in the cytotoxic effects against different cell lines can be thoroughly analyzed, given the similarities in parenthetical structures to determine the future potential target of these flavone derivatives, and may postulate the crucial role of C6-OCH_3_ in combatting prostate cancer. 

In studies targeting PC3 prostate cancer cell lines, both polymethoxyflavones, tangeritin (5,6,7,8,4′-PeMF) and 5-demethyltangeritin (5-hydroxy-6,7,8,4′-TeMF), exerted strong cytotoxicity in a concentration-controlled manner [69]. Within a 48 h treatment, both compounds significantly reduced PC3 cell viability with IC_50_ values of 17.2 and 11.8 µM, respectively (Table 1). The increase in lipophilic magnitude correlates with enhanced hydrophobic capacity and facilitates strong intramolecular hydrogen bonding (IHB) between C5-OH and the neighboring C4 carbonyl group. While IHB was absent with C5-OCH_3_, the methoxy group likely interacts with the target site as HBA, enabling polar interactions with the carbonyl group and the binding target [69]. Conversely, another study on similar cancer cell lines showed minimal bioactivity of nobiletin (5,6,7,8,3′,4′-HeMF) in a concentration-dependent manner with IC_50_ values around 80 µM for the same treatment duration [70]. A comparison of cell viability between the two studies confirmed the inactive cytotoxic effect of nobiletin. Treatment of 20 µM tangeritin reduced cell viability to below 50%, whereas a similar concentration of nobiletin maintained 100% cell viability over the same treatment period. The SAR analysis on prostate cancer cell lines suggests that both hydroxylation and methoxylation at the C5 position contribute to the cytotoxic effects on PC3 cell lines, with few exceptions. The increased bulkiness of ring B in nobiletin with dimethoxy substitutions on ring B at adjacent positions (C3′ and C4′) significantly reduced the hydrogen bonding acidity value, due to the absence of HBD functional groups [45,71,72]. Additionally, the enhanced steric hindrance and elevated partition coefficient (logP) and distribution coefficient (logD) worsen the cytotoxicity capacity [73]. Therefore, the presence of an optimal polar surface provided by hydroxy moieties at ring B introduces an amphipathic characteristic, essential for a more potent cytotoxic effect on cancer cell lines. 

**Figure 6:**
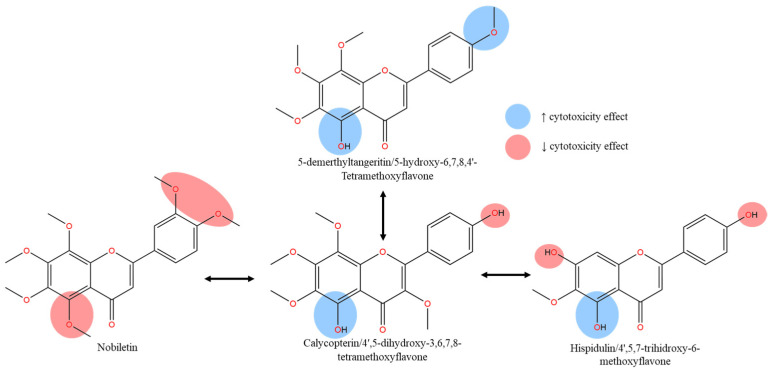
SAR analysis of methoxyflavones analogs and their cytotoxic effects on prostate cancer cell lines. The arrow directions show the transformation from one compound to another, detailing the specific chemical changes like the addition or removal of hydroxyl (-OH) and methoxy (-OCH_3_) groups.

Besides PC3 cell lines, another androgen-independent DU145 cell line is known for its metastatic and invasive behavior due to its non-reliance on hormones for growth [77,78]. Compared to the vehicle group (0.5% DMSO), casticin (5,3′-dihydroxy-3,6,7,4′-TeMF) induced a weak cytotoxic effect maintaining over 80% cell viability at concentrations of 20–50 µM during a 48 h treatment [76]. Structural modifications on rings A and B specifically shifting C4′- to C8-OCH_3_ and C3′-OH to a more favorable hydroxy position at C4′-OH resulted in the creation of calycopterin (5,4′-dihydroxy-3,6,7,8-TeMF). However, the cytotoxic potential of calycopterin remained low with an IC_50_ value of 235 µM in the 48 h treatment [76]. Nevertheless, when comparing the IC_10_ values of both compounds, casticin (IC_10_: 2.5 µM) was significantly more potent than the calycopterin (IC_10_: 20 µM). SAR analysis suggested similar cytotoxic behavior in both hormone-independent PC3 and DU145 cells upon treatment with these derivatives. Multiple in-vitro studies on both prostate cancer cell lines treated with different derivatives demonstrate similar cytotoxic effects, such as curcumin and auraptene [79,80,81,82]. Based on the mechanistic insight, the excessive polar region by the hydroxy group and massive lipophilic effect on ring B would negatively impact the capacity of methoxyflavones analogs to induce cell death. Based on Figure 6, we hypothesized that the absence of at least one methoxy group (C3′ or C5′) adjacent to the C4′-OH adversely reversed the potency. The C6,7-OCH_3_ group was identified as the backbone for the potent cell death effect through lipophilic capacity and pi–alkyl hydrophobic interactions. 

VCaP and LNCaP are androgen-dependent cell lines with weak metastatic potential. Interestingly, owing to the structural similarities of flavonoid families with reproductive hormones, these compounds demonstrate active interactions with androgen receptors [83,84]. The list of phytoestrogenic and phytoandrogenic compounds is known to be polyphenolics which demonstrate strong radical scavengers [85]. Thus, we hypothesize that an increased polar region on ring B of methoxyflavones may induce stronger cytotoxic activity on hormone-dependent cell lines when compared to androgen-independent PC3 and DU145 cancer cell lines. Supporting this hypothesis, calycopterin exhibited potent cytotoxicity on LNCaP cells (IC_50_: 116.5 µM) compared to DU145 cells (235.0 µM) in a 48 h treatment [76], although the IC_50_ still exceeded 100 µM. Similar to the in vitro observations on PC3 cell lines, the influence of C4′-OH in enhancing free radicals scavenging capabilities was suppressed by the IHB formation between C5-OH and the carbonyl group, further limited by the absence of IHB in ring B resulting in a smaller region of electron delocalization [27]. To support the hypothesis, cell viability remained above 80% (100 µM) after the 48 h treatment with nobiletin on VCaP cells, significantly weaker compared to the treatment on hormone-independent PC3 cells [70]. Thus, the lack of a polar region within the aromatic ring B leads to an increased lipophilic capacity, that hinders the hydrogen-donating capability. Likewise, the enormous hydroxylation in the flavone’s lipophilic backbone at ring A in C5,7,4′-OH (hispidulin/5,7,4′-trihidroxy-6-methoxyflavone) failed to improve the cytotoxic effect, proving that the lipophilic effect of methoxy group on ring A is crucial in creating hydrophobic interactions, while hydroxy moieties on ring B elevate HBD and electron delocalization to induced cell death effects [74,86]. We can conclude that both methoxylated and hydroxylated groups play a crucial and synergistic role in their respective region on rings A and B of the flavone structure, maximizing cytotoxic effects on both hormone-dependent and independent cell lines. 

### 2.3. Colon Cancer

**Figure 7:**
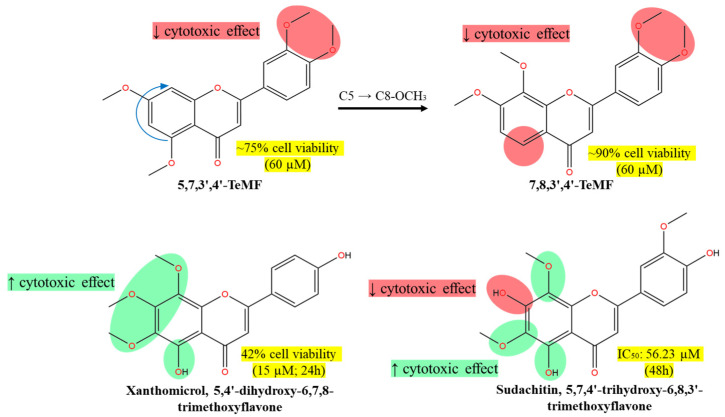
SAR analysis on cytotoxicity activity of methoxyflavones analogs on HCT116 colon cancer cell lines between 24 and 72 h treatment duration [87,88,89]. The molecular structures of various methoxyflavones and their respective impacts on cell viability.

The compound 5,7,3′,4′-TeMF (60 µM) slightly reduced HCT116 cell viability to approximately 75% upon treatment for 72 h [87]. Meanwhile, another tetramethoxyflavones compound, 7,8,3′,4′-TeMF, reduced viability marginally to 90% under the same conditions. The rearrangement of the methoxy group from C5 to C8 did not significantly enhance the cytotoxic effect of the TeMF derivatives on HCT116 cell lines at both positions within the ring A region (Figure 7). The strong hydrophobic effect from the methoxylated substituents, combined with a lack of polar interaction sites on ring B, contribute to the impotent effect. The rearrangement of C5 to C8-OCH_3_ reduced the cytotoxic effect, hypothesizing that C5-OCH_3_ or -OH plays a greater role in influencing the bioactivity of methoxyflavones analogs. Based on the behavior of C8-OCH_3_ on cancer cell lines, these moieties have little to no effect on most anticancer efficacies, with the presence of methoxy group on either the C6 or C7 position. Nevertheless, despite higher cell viability, both tetramethoxyflavones (TeMF) reduced cell migration and invasion, consistently decreasing cell motility and the expression of vimentin and axin2 mRNA, key components of the Wnt-β-catenin signaling pathway in HCT116 cells [87]. 

In vitro treatment of xanthomicrol, 5,4′-dihydroxy-6,7,8-TMF, for 24 h on HCT116 colon cancer cell lines potently reduced the cell viability to 42% at 15 μM and 3% at higher concentrations (21 μM) [88]. Meanwhile, sudachitin, 5,7,4′-trihydroxy-6,8,3′-TMF, moderately suppressed cell viability in HCT116 and HT-29 colorectal cell lines in a 48 h treatment yielding IC_50_ values of 56.23 µM and 37.07 µM, respectively [89]. The screening portrays the hydroxy group at position C5, and C4′ in xanthomicrol was potent against HCT116, while the additional C7-OH in sudachitin disrupted the methoxyflavone hydrophobic and lipophilic scaffold, which moderately impacted the efficacy, owing to stabilized IHB between C3′-OCH_3_ and C4′-OH (Figure 7). As highlighted in our schematic diagram (Figure 3), the disproportionate substitution of the methoxy group, particularly on ring B (C3′ and C4′) of both tetramethoxyflavones leads to steric hindrance that inhibits the formation of intermolecular bonds and reduces the cytotoxic effects. Xanthomicrol and sudachitin demonstrate a perfect hypothesis whereby the weak cytotoxic effect by the extra bulkiness of the methoxylated substituent on ring A and absence of polar region on ring B were offset by hydroxylation of C4′, contributing to stronger bioactivity. The presence of methoxy and hydroxy moieties on position C5 ring A did not significantly influence the bioactivity of flavone derivatives, as both exert similar lipophilic characteristics through different mechanisms—methylation and electron delocalization. Nevertheless, the unexpected cytotoxicity of xanthomicrol on HCT116 gained our interest due to the deviation from the proposed SAR schematic in Figure 3. With the formation of IHB between C5-OH and carbonyl group C4, and the lack of methoxy group on ring B, we are expecting a moderate effect resulting from the exposure of the C4′-OH polar region. For instance, xanthomicrol reported weak activity against HeLa and MCF-7 with an IC_50_ exceeding 100 µM in 24 and 72 h, respectively [47,90]. Meanwhile, calycopterin, with a similar ring B scaffold to xanthomicrol, showed weaker activity against prostate cancer cells [12]. Therefore, we suggest that the HCT116 viable cells may be selectively suppressed by the polar surface of C4′-OH in xanthomicrol indicating a role in cell death induction. The overwhelming cell death induced by xanthomicrol was due to high cell accumulation at the G2/M phase (15 μM) and G1/G0 phase (21 μM) through early and late apoptosis [88]. We believe that C4′-OH may be a critical substituent that triggers the apoptotic cell death pathway, evidenced by its potent IC_50_ values. These findings suggest that increased methoxylation on the flavone skeleton does not correlate to elevated cytotoxicity in cancer cells. Instead, cytotoxicity appears to depend on the specific positioning of substituents and the strategic placement of hydroxyl groups within the flavone structure.

### 2.4. Liver Cancer

Demethylated nobiletin’s derivatives revealed cytotoxic potential against HepG2 liver cancer cell lines [91]. Compound 5,3′-didemethylnobiletin demonstrates the strongest IC_50_ (41.37 μM), followed by 5,3′,4′-tridemethylnobiletin (46.18 μM), 5-demethylobiletin (47.31 μM), and 5,4′-didemethylnobiletin (54.46 μM) when treated for 24 h.

**Figure 8:**
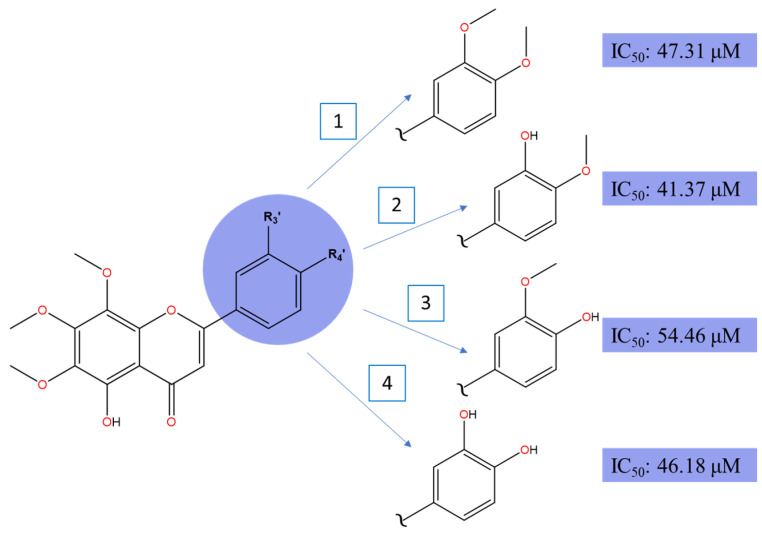
SAR analysis on cytotoxicity activities of 5-demethylnobiletin derivatives on HepG2 liver cancer cell lines in 24 h treatment [91]. Each derivative (1–4) shows a different substitution pattern on the ring B methoxyflavone structure. The IC_50_ values adjacent to each structure illustrate the effect of these modifications on cytotoxic efficacy. Notably, derivative 2 with a hydroxyl group at position R_3′_ shows the lowest IC_50_ value of 41.37 µM, suggesting increased efficacy compared to other derivatives.

Hydroxylation at C5, together with pharmacophore changes between C3′ and C4′, improve cell death effects with C4′ displaying slightly superior potential, as shown in Figure 8. Although the experimental error associated with the IC_50_ values was not provided in the original article, we acknowledge this limitation. However, based on the SAR analysis and mechanistic insights, the observed IC_50_ trends are consistent with the proposed schematic diagram in Figure 3. The cytotoxic potential between C3′- and C4′-OH derivatives narrows down to the target cell death markers, preferrable of the polar surface region on ring B and lipophilic capacity of the target compound. Although C4′-OH flavone derivatives have demonstrated strong cytotoxic activities across various cancer cell lines as compared to C3′-OH with the presence of C5-OH, it may not be the case for HepG2′s anti-cancer mechanisms. In principle, as illustrated in the breast cancer section and multiple studies, the potent effect of C4′-OH is contributed by the greater electron delocalization within rings B and C, which further stabilizes free radicals within the conjugated system and boosts electron donor capability. As a result, the potential of IHB interaction between the adjacent group intensified [45]. To add, C3′ and C4′ interaction with protein targets, through respective electron donor and acceptor capability, could induce more potent cytotoxic effects. A highly stable conformation preserves the molecular structure, but nonetheless, may hinder interactions with the target site, which is coherent with the multiple reports that additional hydroxylation on ring B does not significantly correlate with the potency effect on cancer cells. Interestingly, the interaction that exists between the hydroxy group at C3′ and C4′ on ring B (P4) achieved comparable IC_50_ values to its methoxylated counterpart (P1). To conclude, all four compounds show strong potency despite a variety of functional groups, especially the catechol moiety in ring B, which is commonly found in naturally isolated flavones [92]. Thus, we hypothesize that the demethylation of C5, alongside 6,7,8-trimethoxylated substituent on ring A, is crucial for anticancer activities on HepG2 cell lines. 

**Figure 9:**
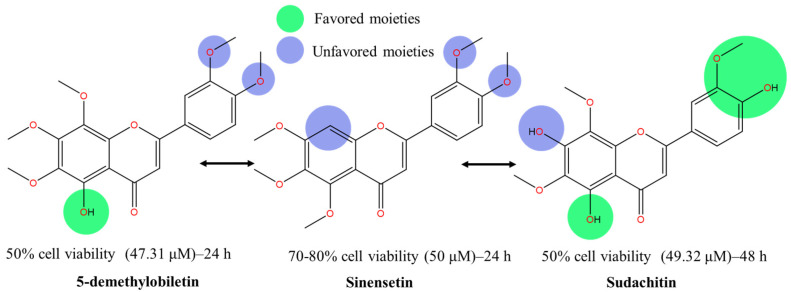
SAR analysis on cytotoxicity activities of 5-demethylnobiletin, sinensetin, and sudachitin analogs on HepG2 colon cancer cell lines for 24 to 48 h treatment [87,91,93]. The arrows indicate structural transformations and their direct impact on cytotoxic efficacy, illustrating how specific modifications in the flavonoid skeleton influence cell death pathways in HepG2 cells.

To validate our hypothesis, the results were compared with an earlier study by Kim et al. [93]. Lipophilic disturbance on ring A, by removal of methoxy and hydroxy groups on C8 and C5, respectively led to a significant drop in cytotoxic effect. In a similar treatment duration (24 h), 50 μM of sinensetin (5,6,7,3′,4′-PeMF) reduced the cell viability by only 30%, with no IC_50_ being reported. Despite a longer treatment period (48 h) being employed, it is worth discussing the cytotoxicity effect of sudachitin due to its unique conformation [87]. While it retained the 5-hydroxy group, justifying a strong IC_50_ (49.32 μM), hydroxylation was also observed on position C7 and C4′ on rings A and B, respectively. Note that as a 24 h treatment was not reported, we assumed the activity of sudachitin might be lower if the cytotoxic effect was in a time-controlled condition. The result, however, appeals to our interest in exploring the activity of C7-OH given its strong cytotoxicity activity on par with the C7-OCH_3_ methoxylated flavone analogs (Figure 9). Our analysis shows current C7-OH methoxylated flavones demonstrate varied cytotoxicity strength against cancer cell lines [74,94]. Acacetin, 5,7-dihydroxy-4′-methoxyflavone, was one of the earlier mono-methoxyflavones studied in anticancer that may untangle the potency of C7-OH. Acacetin reduced cell viability with an estimated IC_50_ of 25 µM after the 24 h treatment [95]. The variation may exist due to the preferential nature of specific moieties and the pathway that leads to the reduction of cell viability. Interestingly, the slight variation in IC_50_ values of the 5-demethylnobiletin may be attributed to the different pathways leading to cell death. The strongest IC_50_ was observed with 5,3′-didemethylnobiletin [91] which induced apoptotic cell death via cleavage on caspase-3 and cell cycle arrest at the G2/M phase. In contrast, its methoxylated counterpart, sinensetin, reduced cell viability by inducing autophagic cell death [93]. With muted expression of pro- and anti-apoptotic markers and early indicators of apoptotic cell death not detected, increased levels of the autophagic marker, beclin-1, and LC3B-II, along with downregulation of p62, confirmed the cell death mechanism of sinensetin. Simultaneous activation of the upstream autophagic marker, p-AMPK, and weakened p-mTOR by sinensetin treatment initiate autophagy cell death.

### 2.5. Acute and Chronic Myeloid Leukemia

FMS-like tyrosine kinase 3 (FLT3) is notably overexpressed in AML, making the inhibition of this kinase a critical focus in leukemia therapy [96,97]. FLT3 activation, facilitated through its extracellular ligand-binding domain, triggers downstream protein cascades that stimulate cell proliferation and differentiation. However, it is a challenge in AML due to the aggressive nature of this cancer. Given the high FLT3 levels, the exploration of flavone derivatives as potential inhibitors is vital. FLT3-internal tandem duplication (FL3-ITD) of positive cell lines such as MOLM-13 and MV4-11 serve as principal models to investigate drug resistance mechanisms against tyrosine kinase inhibitors [98]. An in vitro study by Yen et al. [94] identified four flavone derivatives with potent cytotoxic activity in these cell lines over a 72 h treatment period:

- 5,7-dihydroxy-4′-MF (IC_50_: 9.1 and 6.8 µM);
- 5,4′-dihydroxy-6,7-DMF (IC_50_: 2.6 and 2.6 µM);
- 5,7-dihydroxy-6,4′-DMF (IC_50_: 5.9 and 7.9 µM);
- 5,7,4′-trihydroxy-6-MF/Hispidulin (IC_50_: 7.0 and 6.8 µM).

The compound 5,4′-dihydroxy-6,7-dimethoxyflavone demonstrated the strongest cytotoxic effect, notably due to the preserved hydrophobic properties of C7-OCH_3_ (Table 2). Likewise, substituting the hydroxy group on C7 disrupts the lipophilic capacity with a rich electron delocalization region, which was not favored on ring A. Upon C7 demethylation, pharmacophore changes on C6 and C4′s position are incapable of reversing the cytotoxicity potential. 

In acute and chronic myeloid leukemia, C5-OH has showcased strong evidence as a backbone for an anticancer marker within the flavone class owing to the stabilized IHB with the carbonyl group. Inevitably, researchers are keenly focused on the demethylation of C5 in various 5-methoxyflavones derivatives to leverage the unique intramolecular interactions. Nevertheless, according to Chen et al. [99], 5-demethyl nobiletin exhibited moderate to weak cytotoxic effects against multiple acute myeloid leukemia (AML) cell lines including HL-60, THP-1, U-937, and HEL as well as the chronic myeloid leukemia (CML) cell line, K562. After 48 h of treatment, this compound demonstrated IC_50_ values of 85.7, 32.3, 30.4, and 65.3 μM, respectively, against all AML cell lines. Meanwhile, similar treatment on K562 CML cell lines yielded a slightly weaker IC_50_ value of 91.5 μM. Astonishingly, an earlier study by Yen et al. [100] demonstrated a stronger IC_50_ value of 82.49 μM Despite the favorable C5-OH pharmacophore on multiple cancer cell lines being reported, substitution of dimethoxylation on the C3′ and C4′ position of ring B significantly reduced the hydrogen bond donor capabilities, contributed by diminished electron delocalization by the methoxy group. For nobiletin, under comparable conditions, this suggests that selective binding associated with C5-OCH_3_ could be advantageous as observed in studies involving triple-negative breast cancer, pancreatic (MIA paca-2), and skin cancer cell lines. With limited studies on flavone families, methoxy moieties were reported to be highly favored compared to other cancer cell lines with greater lipophilic character and cytotoxic effect on K562 cell lines [101,102]. Both studies highlighted the contribution of the hydrophobic interaction in facilitating the transport of active compounds to target proteins. Nevertheless, 5,3′-dihydroxy-3,6,7,4′-TeMF, casticin, was investigated for its cytotoxic effects on WEHI-3 mouse leukemia cell lines where it reduced cell viability moderately to 53.4% at a concentration of 1.0 μM for a 48 h treatment, corresponding to an IC_50_ less than 1 μM, markedly more potent than its effects observed on DU145 prostate cancer cell lines [103]. The inhibitory activity reflects the selective characteristic of the flavone compound, which requires high specificity to induce a potent anti-tumor effect by targeting cell death protein markers.

### 2.6. Gastric Cancer

Three polymethoxyflavones, nobiletin, 5-demethyl nobiletin, and tangeritin, have exhibited potent cytotoxic activity against several gastric cancer cell lines including AGS, BGC-823, and SGC-7901 (Table 2) [104]. In a 48 h treatment, tangeritin emerged as the most effective, reducing AGS cell viability to below 20% at an estimated concentration of 33.57 µM. In contrast, 5-demethyl nobiletin showed no activity at concentrations up to 100 µM on both BGC-823 and SGC-7901 cell lines, and 5-demethyl nobiletin was inactive on all tested cell lines. 

Mechanistic and structure-activity relationship (SAR) analysis indicates that the C5-OH group is generally unfavorable against all tested gastric cancer cell lines although showing strong activity in other cancerous cell lines. This inconsistency underscores the need for further pharmacophore studies to fully understand the interactions with the target proteins. The presence of C5-OH was well-known to strengthen the lipophilic capacity of methoxyflavones through IHB, at the expense of diminished C5- moieties interaction with specific protein markers, as discussed in detail in the early section. Interestingly, C5-OCH_3_ appears to maintain stable hydrogen bond interactions as HBA with protein markers by hydrophobic interaction, enhancing the cytotoxic potential of tangeritin significantly. Additionally, the presence of C5-OCH_3_ may facilitate the interaction of the polar carbonyl surface with the target site through an electron transfer/hydrogen acceptor mechanism [105]. Thus, we hypothesize that C5-OCH_3_ and C4 carbonyl pharmacophores hold highly specific importance in initiating strong binding through hydrophobic and polar interaction, with specific proteins that contribute to a stronger cytotoxic effect. On the other hand, the heavy substitution of the methoxy group on ring B does diminish the cytotoxicity capacity of nobiletin compared to tangeritin, in line with our analysis based on Figure 3. The mechanism demonstrates how specific functional groups significantly affect the binding interaction and overall anticancer efficacy of these compounds. 

### 2.7. Skin Cancer

Among four methoxyflavones isolated from *Gardenia oudiepe* (Table 2), only two PMFs displayed a strong cytotoxic effect against A2058 melanoma cell lines [106]. Treatment with compounds 5,7-dihydroxy-3,6,4′-TMF (**P1**) and 5,7,5′-trihydroxy-3,6,3′,4′-TeMF (**P2**) at 10 µM for 72 h significantly reduced cancer cell growth by 66.52% and 42.86%, respectively, yielding IC_50_ of 3.92 and 8.18 µM, respectively. Meanwhile, both compounds 5,7,4′-trihydroxy-3,6-DMF (**P3**) and 5,7-dihydroxy-3,6,3′,4′,5′-PeMF (**P4**) showed the least cytotoxic potential due to amphipathicity disruption on ring B. The A2058 melanoma cell lines are highly invasive and metastasized, and having extra lipophilic and hydrophobic characteristics on ring B was expected to improve the cytotoxic effect of the methoxyflavones analogs. 

The substitution of the methoxy group (**P1**) with hydroxy group (**P4)** at position C4′ reduced the hydrophobic strength on ring B and diminished its growth inhibition effect significantly from 66.52% to 18.55% under the same concentration and treatment duration. Nevertheless, the neighboring effect of a methoxy group (C4′) and a hydroxyl group (C5′) may have triggered the formation of IHB, stabilizing the compound and improving its hydrophobic capacity with the addition of C3′-OCH_3_ (**P2**) despite the intense bulkiness within ring B. On another note, additional C5′ methoxy (5,7-dihydroxy-3,6,3′,4′,5′-PeMF) replacing the hydroxy group reversed the cytotoxic effect and significantly reduced its effectiveness with >90% cancer cell viability. The massive hydrophobic effect introduced by the addition of a trimethoxy group on ring B without possible interaction from the adjacent functional group is the main contributing factor to its reduced efficacy. However, both compounds **P1** and **P2** induced potent inhibitory effects with strong IC_50_ values of 3.92 and 8.18 μM, respectively, through an apoptotic cell death mechanism involving the cleavage of caspase-3.

### 2.8. Oral Cancer

Five methoxyflavone compounds isolated from *Hottonia palustris* were tested on SCC-25 squamous carcinoma cell lines over 24 and 48 h demonstrating distinct cytotoxic activity [107]. Of these, only one isolated PMF (5,6′-dihydroxy-2′,3′-DMF) showed moderate cytotoxic effects with IC_50_ values of 78.2 and 40.6 µM, on respective duration treatment. Meanwhile, 5-hydroxy-2′-MF, 5-hydroxy-2′,6′-DMF, 5-hydroxy-2′,3′,6′-TMF, and 5,2′-dihydroxy-6′-MF were ineffective with IC_50_ values exceeding 200 µM (Table 2). 

Mechanistic and SAR analysis suggests that a balance between both hydrophobic and polar interactions across rings A and B is crucial. Based on the screening, these compounds are notable among flavone derivatives for having methoxy or hydroxy groups bonded to the C6′ position on ring B. Based on the cytotoxic effect, it is interesting to highlight the structural similarities between 5,6′-dihydroxy-2′,3′-DMF (IC_50_: 40.6 µM) and 5-hydroxy-2′,3′,6′-TMF (IC_50_ > 200 µM). Position C6′ was hypothesized to significantly influence the bioactivity of the isolated PMF on SCC-25 cell lines. Early screening demonstrates the favorable C6′-OH position on ring B that provides an alternative position to maximize the polar region, without significant disruption on lipophilic capacity by C5-OH and methoxy group on ring A. Nevertheless, more analysis is required to confirm the pharmacophore of C6′ moieties that contributes to a strong cytotoxicity effect. Additionally, except for compound 5,6′-dihydroxy-2′,3′-DMF, hydroxylation on ring B was not observed in the other screened methoxyflavones, which is crucial to the enhanced resonance effect of free radicals. As illustrated in Figure 4, the strong activity of C6′-OH may be attributed to the maximized free radical’s delocalization between ring B and C, that is offset by the greater lipophilic effect of dimethoxy substitution in the neighboring position (C2′ and C3′).

### 2.9. Bile Duct and Pancreatic Cancer

In bile duct cancer, sudachitin (5,7,4′-trihydroxy-6,8,3′-TMF) significantly reduces cancer cell viability with lower IC_50_ values of HuCCT1 (53.21 µM) and RBE (24.1 µM) in a 48 h treatment [89] (Table 2). As discussed previously, sudachitin is well known for its potent activity in various cancer types, including colon and pancreatic cancers. Nevertheless, the detailed cell death mechanisms have remained scarce and have not been investigated up to the recent studies. In contrast, nobiletin (5,6,7,8,3′,4′–HeMF) is unable to reduce cell viability in both TFK1 and RBE cell lines, even at concentrations up to 100 µM in both 24 and 48 h treatments [108]. As previously discussed, the inactivity of nobiletin against certain cancer cell lines, including breast and prostate cancer cell lines, is attributed to its massive lipophilic effect and selective cell death pathways. The GSK3β pathway is identified as contributing to nobiletin’s antiproliferative effects based on Western blot analysis. Resembling the bile duct cancer, sudachitin shows potent activity against MIA PaCa-2 and PANC-1 pancreatic cancer cell lines, in 48 h treatment (IC_50_: 43.35 and 32.73 µM) [89]. Meanwhile, nobiletin displays weak cytotoxicity effects on these cells with PANC-1 cell viability remaining above 80% even after treatment at the highest concentration (120 μM) and longest duration treatment (72 h) [109]. Nonetheless, nobiletin shows significant cytotoxicity effects of MIA PaCa-2 cells after a 72 h treatment, reducing cell viability to less than 20% at 80 μM. The selective cytotoxicity of nobiletin may be attributed to the heterogeneity of the cells, with PANC-1 being reported to be more invasive and have a greater metastatic effect than MIA PaCa-2 cells. 

### 2.10. Cervical Cancer

The growth of cervical cancer cell lines has been associated with human papillomaviruses (HPVs) infections, notably its subgroup HPV18+ (Hela) and HPV16+ (C33A). Meanwhile, SiHa cells are HPV negative (HPV-) [110]. The HPV oncoproteins E6 and E7 are known to facilitate malignant tumor conversion by disrupting the cancer cell death markers, which is evident in HPV18+ Hela cells [111,112]. In both studies reported by Zhang et al. [113,114], compound 6-methoxyflavone exhibited potent cytotoxicity against cervical cancer cells. Over a 72 h treatment period, this compound reduced Hela cell viability the most (IC_50_: 55.31 µM), followed by C33A (109.57 µM and SiHa (208.53 µM) (Table 2). Although compound 6-methoxyflavone was the most potent to Hela, its IC_50_ value was considered moderate, compared to the other cancer cell lines. Interestingly, a more complex methoxyflavones, 4′,5′-dihydroxy-5,7,3′-TMF, shows a strong cytotoxic effect on Hela cells in a similar 72 h treatment (IC_50_: 4.83 µM). A similar compound has shown a strong cell death effect on breast and ovarian cancer cell lines with IC_50_ less than 20 µM [64]. Meanwhile, another methoxyflavones, xanthomicrol, 5,4′-dihydroxy-6,7,8-TMF, was less potent against Hela cells with an IC_50_ exceeding 100 µM in a 72 h treatment [90]. Having at least a single methoxy group on ring A (C6/7/8-OCH_3_) has shown promising anticancer potential by preserving the hydrophobic capacity of the analogs. The addition of a single methoxy group (C3′) next to the hydroxy group (C4′ and C5′) of compound 4′,5′-dihydroxy-5,7,3′-TMF creates an enormous IHB formation capability and appropriate polar region for HBD interaction with the target site, as discussed previously on Section 2.1 and Section 2.2. Thus, the absence of a methoxy group on ring B of xanthomicrol may destabilize both symbiotic relationships of the polar and lipophilic region of the respective compound, resulting in a weaker effect. With that, chrysoplenetin, 5,4′-dihidroxy-3,6,7,3′-TeMF, having a similar structural pharmacophore to xanthomicrol and fulfilling the specific conditions, significantly improved the cytotoxic activity in a shorter treatment duration of 48 h with IC_50_ value of 53 µM [50,115].

### 2.11. Ovarian Cancer

With limited studies utilizing methoxyflavones scaffold, the SAR profiling is limited to targeting ovarian cancer cell lines. Based on Table 2, treatment with wogonin on all cisplatin-sensitive (SKOV3 and OV2008) and its respective resistance counterpart (SKOV3/DDP and C13*) ovarian cancer cell lines for 72 h at doses up to 20 µM demonstrated a weak cell death effect with cell viability remaining above 80% [116]. Nonetheless, at the highest concentration (160 µM), wogonin significantly reduced all cell viabilities to 20%. Whereas, similar to the breast and cervical cancer cell line, compound 4′,5′-dihydroxy-5,7,3′-TMF demonstrated the strongest IC_50_ value of 10.64 µM, followed by 5,7,3′,4′,5′-PeMF (IC_50_: 30.17 µM) and 5,6,7,8,3′,4′,5′-heptamethoxyflavone (IC_50_: 63.04 µM) on SKOV3 cancer cell lines [64]. The absence of a hydroxy group as a source for HBD on ring B tripled the IC_50_ level. Meanwhile, greater methoxy substitution strongly correlates with extreme lipophilic and weakening cytotoxicity effects, which doubled from 30.17 to 63.04 µM, respectively. 

## 3. Mechanism of Methoxy and Hydroxy Flavones Derivatives

### 3.1. Flavones Modulate the Apoptotic Cell Death Pathway

Briefly, recent studies on the antitumor mechanism of flavone derivatives depict a multi-strategy approach. These compounds leverage the versatile positions available for hydroxy and methoxy substituents in flavones’ molecular structure, influencing various carcinogenic pathways. Apoptosis or programmed cell death has been a hallmark mechanism employed by flavones to prevent oncogenesis through both extrinsic and intrinsic mitochondrial pathways. The activation of mitochondrial outer membrane permeabilization (MOMP) is crucial in stimulating the expression of apoptotic proteins. Caspase-3, a critical player in apoptosis for methoxyflavones derivatives, is often due to its central role in both mitochondrial pathways that lead to cell death [40,117,118,119]. For instance, nobiletin [104] and 6-methoxyflavone [113,114] induce apoptosis via the extrinsic or death receptor pathways, activating receptors such as Fas and tumor necrosis factor (TNF). After the caspase-3 cleavage, the upregulation of tumor suppressor p53 in the intrinsic mitochondrial pathway occurred due to mutation in the Tp53 gene, given its primary roles in cell cycle arrest, DNA repair, and apoptosis. Based on the literature flavones derivatives, xanthomicrol [88], 5,3′-dihydroxy-3,6,7,8,4′-PMF [48], tangeritin [104], 5-demethylnobiletin [104], 5,3′-didemethylnobiletin [91], 5,4′-didemethylnobiletin [91], and 5,3′,4′-tridemethylnobiletin [91] activate the Bcl-2 family’s protein, which comprises pro-apoptotic, anti-apoptotic, and BH3-only proteins, controlled by the expression level of p53 [93,114,120,121]. Elevated levels of pro-apoptotic proteins such as BID, BAX, and BAK alongside the downregulation of anti-apoptotic proteins, MCL-1, Bcl-2, and Bcl-xL in the intrinsic mitochondrial pathway resulted in activation of caspase-3, -8, and -9, followed by apoptotic cell death [48,103]. In some cases, the variation of pro- and anti-apoptotic protein expression levels induced the release of cytochrome C, subsequently inhibiting APAF-1 mRNA expression before caspase cleavage, as observed on the Hela cell line after being treated with 6-methoxyflavone [113,114]. 

### 3.2. Flavones-Induced Cell Cycle Arrest

Flavones not only induce apoptosis but also impact cell cycle regulation through the tumor suppressor protein p53. The transcription factor p53 is a central tumor-blocking protein that activates dozens of downstream genes that modulate cell cycles. Numerous studies have illustrated the affiliation of p53 with its transcriptional target, p21^Waf1/CIP1^ in suppressing cyclins and cyclin-dependent kinase (CDK) expression by forming inhibitory complexes that lead to cell cycle arrest [122,123]. Flavone derivatives such as xanthomicrol [88] activate p21^Waf1/CIP1^ via the transcriptional target of the P53 arrest cell cycle at the G0/G1 phase, consequently inhibiting cyclin D and CDK4 expression. Other methoxyflavones including nobiletin [108], 5-demethyltangeritin, acacetin [94], pectolinarigenin [94], hispidulin [94], and 5,4′-dihydroxy-6,7-DMF [94] suppressed the cell cycle in a similar G0/G1 phase. Concurrently, Lotfizadeh et al. [76] and Yen et al. [100] depict similar behavior of calycopterin and nobiletin, respectively, by inducing cell cycle arrest at the G1 phase, with nobiletin specifically activating p21 and p27 expression, and suppressing cyclin D mRNA expression. Nevertheless, by modifying nobiletin by replacing the methoxy group with hydroxy at the C5 position, compound 5-demethylnobiletin [99] notably influences the cell cycle arrest, shifting the cell cycle arrest towards the G1/S phase with weaker expression of cyclin E and A mediated by p21. In another study, 5-demethylnobiletin derivatives [91] and an isolated compound from *Gardenia oudiepe*, 5,7-dihydroxy-3,6,4′-TMF [106] capable of inhibiting the cell cycle at the G2/M phase leads to apoptosis. Meanwhile, 6-methoxyflavone [113,114] depicts cell cycle arrest at the S phase through p21^Waf1/CIP1^ activation resulting in the suppression of cyclin A and CDK2 activities.

### 3.3. Mitogen-Activated Protein Kinase (MAPK) Pathway

The mitogen-activated protein kinase (MAPK) signaling pathway, centered around the RAS oncogene GTP-binding protein, has been targeted for colorectal and pancreatic cancer therapies since the late 1970s [124,125]. The pathway involves the inhibition of the RAS upstream novel target, SOS-1, a guanine nucleotide exchange factor. This inhibition prevents the conversion of GDP to GTP by RAS assisted by GRB2 (growth factor receptor bound protein 2), thus blocking RAF dimerization and subsequent downstream signaling [126]. Over 85% of cancers are marked by abnormal activity of MAPK, emphasizing the need for targeted intervention by suppressing the mitogen-activated proteins. The activation of SOS-1 triggers a series of interactions among RAF, MEK, and ERK1/2, resulting in the abnormal activation of the MAPK pathway [127]. Within the cytosol, casticin [75] has been shown to inhibit both SOS-1 and GRB2, reducing phosphorylation levels of ERK1/2, a downstream component of the MAPK pathway, indicating suppression of MAPK signaling [70,75], whereas nobiletin [70] targets a similar cell death mechanism by reducing the phosphorylation levels of ERK1/2. This inhibition also leads to suppression of c-myc, a transcription factor with a positive correlation with this pathway [128]. Moreover, while regulating MAPK signaling, RAS and GRB2 are well-known to activate the PI3K/Akt pathway, contributing to oncogenic behavior and cancer progression [129]. Stimulation of both oncogene-induced RAS-activated PI3K together with the amplification of mutation on its major effector, AKT and mTOR drives the malignant resistance and tumor progression [130]. Methoxyflavones derivatives like wogonin [116], sinensetin [93], and casticin [75] are noted for inhibiting angiogenesis by impacting these critical pathways, highlighting their potential in therapeutic strategies against cancer.

### 3.4. NF-κB Signaling

NF-κB, a key transcription factor, plays a critical role in inflammation and cancer by promoting tumorigenesis through the activation of various genes including IκB, p65, and p50. This pathway, involved in both canonical and non-canonical pathways, is crucial for DNA repair, drug resistance, inhibition of apoptosis, and promotion of cell proliferation [131,132]. Various studies consistently link NF-κB to cancer progression, notably its role in resisting TNF-α-induced apoptosis by enhancing ROS levels and antioxidant enzyme activities, thereby preventing cell death [133]. 

Nobiletin [65], a methoxyflavone derivative, has been identified as an anti-inflammatory agent that can moderate the activation of NF-κB triggered by LPS- and TNF-α as demonstrated by numerous studies [134,135]. Concurrent investigation on the activity of the PARP-1 nuclear enzyme was important given its roles in DNA repair and reversing cancer cell death by triggering NF-κB expression [136]. For instance, the inhibition of PARP-1 by 5-demethylnobiletin in AML cell lines has been shown to promote apoptosis, effectively leading to leukemic cancer cell death as discussed by Chen et al. [99]. Meanwhile, phosphorylated NF-κB (p-p65) were markedly downregulated in LPS-induced cancer cells when treated with nobiletin [60] while TNF-α-induced NF-κB expression was suppressed with upregulation of IκBα. Nonetheless, treatment with flavones has been associated with an overexpression of PARP-1 which suggests a potential unique mechanism of action. This overexpression leads to an increase in Poly(ADP-ribosyl)ation (PAR) synthesis during early apoptosis, subsequently activating caspase-3 and initiating the proteolysis of PARP-1, which contributes to the strong cytotoxic effects [137].

### 3.5. Matrix Metalloproteinase (MMP) Cascades Signaling

Cancer-associated fibroblast (CAF) is one of the largest groups in the matrix component and part of the tumor microenvironment, and plays a crucial role in cancer progression [138]. These cells, when activated from normal fibroblast within adipose tissue notably by cancer cells such as the 4T1 breast cancer cell line, exhibited increased mRNA expression of fibroblast biomarker, alpha-smooth muscle actin (α-sma), vimentin, and others [138]. This interaction between CAFs and tumor cells contributes to the micro-interaction between the CAF and tumor cells and contributes to the development of highly metastatic and invasive cancer cells. 

Research has shown that PMF, specifically oroxylin A, successfully reduced the mRNA expression of α-sma, vimentin, and fibronectin without significant toxicity in killing normal cell L-929 and normal primary cell, NBF, indicating selective efficacy. Furthermore, CAF activation leads to higher invasiveness in the 4T1 cells, of which oroxylin A has been potently downregulating the mRNA expression of MMP-2, MMP-9, and MMP-14 in a dose-controlled manner. The inactivation of CAF by PMF is facilitated by targeting ACTN1 through interaction at ASP-781, as revealed by molecular docking analysis. This interaction leads to a significant reduction in the phosphorylation of downstream signaling pathways, including focal adhesion kinase (FAK) and janus kinase 2 (JAK2), which was the main target market for acacetin [95] in the human hepatocellular carcinoma cell line, HepG2. Additionally, the multi-target roles of nobiletin [109] include the suppression of the mammalian target of rapamycin (mTOR), and signal transducer and activator of transcription 3 (STAT3) phosphorylation, thereby inhibiting key processes that promote PANC cancer cell migration and invasion.

**Table 2:** Cytotoxicity assay of methoxyflavones analogs. Bold IC_50_ refers to strong cytotoxic activity.

Compound	Natural Sources	Cancer	Cells	Treatment (hr)	IC_50_ (µM)	References
5,3′-dihydroxy-3,6,7,8,4′-PeMF	*Glycosmis ovoidea Pierre*	Breast	MCF-7	24	49.46 µM	[48]
				48	17.56 µM	
				72	**3.71 µM**	
			MDA-MB-231	24	49.46 µM	
				48	49.46 µM	
				72	21.27 µM	
Nobiletin	*Citrus sinensis*/*Citrus reticulata*	Gastric	AGS	48	>12.5 mg/L (>31.06 µM)	[104]
			BGC-823		>50 mg/L (>124.26 µM)	
			SGC-7901		>50 mg/L (>124.26 µM)	
		CML	K562	48	82.49 µM	[100]
		Prostate	VCaP	48	>120 µM	[70]
			PC3	48	80–100 µM	
		Pancreas	MIA PaCa-2	72	>40 µM	[109]
			PANC-1	72	>120 µM	
		Breast	MDA-MB-231	48	>30 µM	[60]
		Bile duct	TFK1	48	Inactive	[108]
			RBE	48	Inactive	
5-demethylnobiletin	*Citrus reticulata*	Liver	HepG2	24	47.31 µM	[91]
		AML	HL-60	48	85.7 µM	[99]
			THP-1		32.3 µM	
			U-937		30.4 µM	
			HEL		65.3 µM	
		CML	K562		91.5 µM	
		Gastric	AGS		>100 mg/L (>257.49 µM)	[104]
			BGC-823		>200 mg/L (>514.97 µM)	
			SGC-7901		>50 mg/L (>128.74 µM)	
5,7,4′-trihydroxy-6,8,3′-TMF/sudachitin	*Citrus sudachi*	Colon	HCT116	48	56.23 µM	[89]
			HT-29	48	37.07 µM	
		Liver	HepG2	48	49.32 µM	
		Bile duct	HuCCT1	48	53.21 µM	
			RBE	48	24.1 µM	
		Pancreas	MIA PaCa-2	48	43.35 µM	
			PANC-1	48	32.73 µM	
5,6′-dihydroxy-2′,3′-DMF	*Hottonia palustris*	Oral	SCC-25	24	78.2 µM	[107]
				48	40.6 µM	
5-hydroxy-2′-MF					Inactive	
5-hydroxy-2′,6′-DMF					Inactive	
5-hydroxy-2′,3′,6′-TMF					Inactive	
5,2′-dihydroxy-6′-MF					Inactive	
5,7-dihydroxy-4′-MF/acacetin	*Robinia pseudoacacia*		MOLM-13	72	**9.1 µM**	[85]
5,4′-dihydroxy-6,7-DMF	*Quercus incana*				**2.6 µM**	
5,7-dihydroxy-6,4′-DMF/Pectolinarigenin	*Artemisia capillaris*				**5.9 µM**	
5,7,4′-trihydroxy-6-MF/hispidulin	*Artemisia capillaris*				**7.0 µM**	
5,7,4′-trihydroxy-6-MF/hispidulin	*Artemisia capillaris*		MV4-11		**6.8 µM**	
5,4′-dihydroxy-6,7-DMF	*Quercus incana*				**2.6 µM**	
5,7-dihydroxy-6,4′-DMF/Pectolinarigenin	*Artemisia capillaris*				**7.9 µM**	
5,7-dihydroxy-4′-MF	*Robinia pseudoacacia*				**6.8 µM**	
5,7-dihydroxy-3,6,4′-TMF	*Gardenia oudiepe* (Rubiaceae)	Skin	A2058	72	**3.92 µM**	[106]
5,7,5′-trihydroxy-3,6,3′,4′-TeMF					**8.18 µM**	
5,7,4′-trihydroxy-3,6-DMF					Inactive	
5,7-dihydroxy-3,6,3′,4′,5′-PeMF					Inactive	
5,7-dihydroxy-8-MF/wogonin	*Scutellaria baicalensis*	Ovarian	SKOV3	72	<40 µM	[116]
			OV2008		40–80 µM	
			SKOV3/DDP			
			C13*			
4′,5′-dihydroxy-5,7,3′-TMF	Synthetic compound	Breast	HCC1954	72	**8.58 µM**	[64]
		Cervical	Hela		**4.83 µM**	
		Ovarian	SKOV3		**10.64 µM**	
4′-hydroxy-5,7,3′,5′-TeMF			HCC1954		>100 µM	
			Hela		>100 µM	
			SKOV3		>100 µM	
5,7,3′,4′,5′-PeMF			HCC1954		53.84 µM	
			Hela		40.82 µM	
			SKOV3		30.17 µM	
5,6,7,8,3′,4′,5′-heptamethoxyflavone			HCC1954		>100 µM	
			Hela		35.80 µM	
			SKOV3		63.04 µM	
5,6,7,8,4′-PeMF/tangeritin	*Citrus reticulata*	Prostate	PC3	48	**17.2 µM**	[69]
		Gastric	AGS	48	<12.5 mg/L (<33.57 µM)	[104]
			BGC-823	48	<50 mg/L (<134.28 µM)	
			SGC-7901			
5,4′-dihydroxy-6,7,8-TMF/xanthomicrol	*Achillea erba-rotta* subsp. *moschata*	Colon	HCT116	24	**<15 µM**	[88]
		Breast	MCF7	72	>100 µM	[49]
		Cervical	Hela	24	>100 µM	[90]
5,3′,4′-trihydroxy-6,7,8-TMF/sideritoflavone		Breast	MCF-7	72	**4.9 ± 1.7 μM**	[47]
		Breast	JIMT-1	72	**1.9 ± 0.3 μM**	
6-MF	*Pimelea decora*	Cervical	Hela	72	55.31 ± 9.14 µM	[113,114]
			C33A	72	109.57 ± 4.05 µM	
			SiHa	72	208.53 ± 10.96 µM	
5,3′-didemethylnobiletin	*Citrus reticulata*	Liver	HepG2	24	41.37 µM	[91]
5,4′-didemethylnobiletin					54.46 µM	
5,3′,4′-tridemethylnobiletin					46.18 µM	
5,7,3′,4′-TeMF	*Kaempferia parviflora*	Colon	HCT116	72	>60 µM	[87]
7,8,3′,4′-TeMF	*Citrus reticulata*			72	>60 µM	
5,4′-dihydroxy-3,6,7,8-TeMF/calycopterin	*D. kotschyi Boiss*	Prostate	DU145	48	235 µM	[76]
			LNCaP	48	116.5 µM	
5,4′-dihydroxy-6,7-DMF/cirsimaritin	*Quercus incana*	Lung	NCI-H460	48	26.23 ± 0.053 µM	[120]
5,3′-dihydroxy-6,7,4′-TMF/eupatorin					37.50 ± 0.070 µM	
5,7-dihydroxy-4′-MF/acacetin	*Robinia pseudoacacia*	Liver	HepG2	24	>25 µM	[95]
				48	>15 µM	
				72	>5 µM	
5,6,7,3′,4′-PeMF/Sinensetin	*O. aristatus leaves*		HepG2	24	>100 µM	[93]
				48	<50 µM	
5,7-dihydroxy-6-MF/Oroxylin A	*Scutellaria baicalensis*	Breast	4T1	24	Inactive	[138]
5,7,4′-trihydroxy-6-MF	*Artemisia capillaris*	Prostate	VCaP	24	>100 µM	[74]
				48	>50 µM	
			DU145	24	>1000 µM	
				48	>50 µM	
5,4′-dihidroxy-3,6,7,3′-TeMF/chrysosplenetin	*Artemisia annua* L.	Breast	MCF-7	72	**0.3 µM**	[50]
		Cervical	HeLa	48	53 µM	[50,115]
		Breast	MCF-7	48	**4.2 µM**	
5-hydroxy-6,7,8,4′-TeMF/5-Demethyltangeritin	*Citrus reticulata*	Prostate	PC3	48	**11.8 µM**	[69]
5,3′-dihydroxy-3,6,7,4′-TeMF/casticin	*Dracocephalum kotschyi Boiss*	Prostate	DU145	48	>50 µM	[75]

### 3.6. Autophagy in Cancer

Autophagy, a self-degradation process essential for removing damaged organelles and other intracellular components, involves the formation of autophagosomes that fuse with lysosomes for decomposition and recycling [139]. In cancer, autophagy plays a dual indefinite role: it suppresses cancer growth in the earlier stage by eliminating defective components that could promote malignancy, and it may support tumor growth at later stages by aiding cancer cell survival under stress [140].

One of the critical functions of autophagy in cancer is its ability to prevent the accumulation of ROS that leads to oxidative stress and DNA damage. This prevention is achieved by blocking the lipidation of LC3 protein, converting it from LC3-I to LC3-II via ATG4, an enzyme essential for autophagosome formation [141,142]. An extensive review by Zhao et al. [143] discussed the regulatory roles of the mTOR-ATG4- LC3-II pathway in regulating ROS levels and autophagy. A study by Wang et al. [74] explored the potential antiproliferative effects of hispidulin, a compound known to influence this pathway, on DU145 and VCaP prostate cancer cell lines. Hispidulin potency reduced cell migration and invasion after a 48 h treatment, suggesting its potential to elevate autophagy in these cancer cells. Evidence of an increased rate of autophagy flux, characterized by the conversion of LC3 I to LC3 II, and elevated PPARγ expression, in tandem with weak phosphorylation of mTOR and S6K1, supports the hypothesis that hispidulin can modulate autophagy to suppress tumor progression. 

### 3.7. ER-Stress-Induced Apoptosis by Flavone

The progression of malignant tumors is often influenced by the stress induced within the cells intrinsically and extrinsically resulting in disruption within the tumor microenvironment [144]. One significant source of cellular stress is the endoplasmic reticulum (ER), where irregularities and disruptions in protein folding can activate stress-induced signaling pathways. These pathways not only promote cancer cell growth but also enhance metastatic potential [145,146]. A key component in the ER stress response is the PERK signaling pathway which plays a vital role in mediating apoptosis through the mitochondrial pathway via BAX/Bcl-2 regulation or through triggering autophagy by activating downstream markers. For instance, 6-methoxyflavone [113,114] induces autophagy through elevated mRNA expression of downstream proteins such as PERK, EIF2α, IRE1α, ATF4, and CHOP. This enhanced autophagic response to ER stress presents a significant mechanism by which flavones may exert their anticancer effects, providing a dual approach to controlling tumor progression through both apoptosis and autophagy.

### 3.8. Targeting Topoisomerase Enzymes in Highly Expressed Cancer Cells

Topoisomerases I and II are critical enzymes in DNA replication and transcription, and they are often highly expressed in various cancers including breast cancer [147]. Methoxyflavones derivatives have been identified as potent inhibitors of these enzymes exploiting their ability to interfere with the unwinding process of DNA, leading to cell death [148]. For instance, chrysosplenetin [50], a specific methoxyflavones derivative, has shown a strong inhibitory effect on topoisomerase I. The strong potency of the compound may be attributed to the targeting inhibition of the topoisomerase enzyme. The strong inhibitory effect of chrysosplenetin toward topoisomerase I leads to a higher form I percentage in a concentration-dependent manner. The Form I of pBR322 plasmid DNA increased from 31.0% to 69.0%, whereas the percentage of Forms II and III declined. Meanwhile, topoisomerase II was inhibited by the compound in a similar concentration where Form I accumulation increased to 96.9% at the highest concentration. These findings highlight the potent anticancer properties of methoxyflavones derivatives through their targeted action against topoisomerase enzymes providing a promising avenue for therapeutic intervention in cancers with high topoisomerase expression.

### 3.9. Wnt-β-Catenin Pathway

The Wnt-β-catenin signaling pathway is critically involved in uncontrolled cancer cell proliferation, especially noted in colorectal cancer cells. Abnormalities in this pathway contribute to the cancer’s invasive and metastatic characteristics [149]. Dysregulation of the key signaling cofactor β-catenin is known to influence the development of a cancer stem cell phenotype, characterized by high levels of cyclin D and c-myc, which are crucial for tumorigenesis [150]. The activation of this pathway leads to the elevated expression of downstream Wnt target genes, which include axin2 and vimentin which drive the cancer progression [151]. Western blot assay on sideritoflavone [47] activates Wnt, Myc/Ma, and transforming p64/NF-κB expression. Studies using the RT-PCR have shown that tetramethoxyflavone specifically 5,7,3′,4′-TeMF [87] and 7,8,3′,4′-TeMF [87] significantly downregulate the mRNA expression of c-Myc, axin2, and vimentin in HCT116 colorectal cells at a concentration of 30 µM. 

## 4. Summary

The multi-target of methoxyflavones has contributed to various biological activities in recent in vitro studies. Being naturally abundant, the flavonoid subclass could exist in a variety range of scaffolds with distinct features. Our review indicates that major parts of the methoxyflavones scaffold have not been fully explored with limited findings. The pharmacophore of the anticancer effect of methoxyflavones was centered on ring A, strengthened by C3′ and C4′ ring B, with few compounds bearing C2′, C5′, and C6′ moieties (Figure 3). The superiority of the anticancer effect of methoxyflavones against other natural flavones such as hydroxyflavones and the flavones glycosides was established. The methoxy group governed the lipophilic capacity and facilitated drug-membrane transfer, thus preserving the bioavailability of the scaffolds. The lipophilic character of methoxyflavones was the main contribution to a remarkable activity against various cancer cell lines, based on our analysis. Additionally, critical investigation summarized that the methoxyflavones themselves could not sustain a stronger cytotoxic effect of IC_50_ less than 20 µM without the presence of the hydroxy group. 

The preservation of lipophilic properties on ring A flavones, with a balance capacity between a polar surface and lipophilic effect on ring B, is the pathway to achieve stronger IC_50_. Generally, methoxy moieties occupied at least two out of three positions from the C6, 7, and 8 positions, with C7-OCH_3_ anchored to the strong cytotoxic activity of methoxyflavones analogues. Alternatively, C7-OH could exert a similar effect with the addition of at least one C6-OCH_3_ group. The addition of a hydroxy group on position C5 initiated a formation of intramolecular hydrogen bonding with the C4 carbonyl group, fundamental to stronger anticancer activity, with few conditions. Next, having hydroxy moieties alongside the methoxy group on either the C3′ or C4′ arrangement could amplify the cytotoxicity activity by the resonance effect of free radicals which contribute to greater hydrogen bond donor capacities. Both methoxy or hydroxy groups in the respective position on ring B could achieve similarly strong IC_50_, yet it came down to the conformational changes on rings A and B and on the cell types. Some C5-OCH_3_ derivatives could induce strong IC_50_, with few exceptions such as a maximum of a single hydroxy or methoxy group on ring B. Excessive substitution of the methoxy group on ring B significantly deteriorates the IC_50_ of the scaffold on numerous cancer cell lines, either on C5-OH or C5-OCH_3_ methoxyflavones analogs [71,72]. The absence of hydroxy moieties on ring B could be offset by a single methoxylated substitution on the same ring, for instance, 5-demethyltangeritin, although the activity may subdue [69]. To maximize interaction with the target protein marker, balancing between the methoxy and hydroxy moieties in terms of the number and position of both groups in ring A and B flavones is crucial. Additionally, the collaborative efficacy of both groups will maximize the resonance effect and electron delocalization of free radical flavones and initiate the formation of IHB. Concurrently, both interaction mechanisms stabilized and preserved the lipophilicity while simultaneously offering an optimal polar surface for stronger interaction with the cancer cell death marker. The review solely focused on the pharmacophore specificity of methoxyflavones analogs in vitro, with other factors such as drug metabolism, bioavailability, and toxicity, which may influence the resultant efficacy. This review may narrow down future research for targeting and isolating active natural methoxyflavones and assisting in the synthetic designing of flavones scaffolds with favorable positions of both methoxy and hydroxy moieties.

## Figures and Tables

**Table 1:** Comparative Cytotoxic Effects of Methoxyflavones against Prostate Cancer Cell Lines. The pharmacophore changes on ring B flavones (C3′ and C4′) significantly influenced the IC_50_ of the analogs. Green indicates favorable moieties and red signifies an unfavorable position.

Prostate Cancer Cell Lines	C_3_	C_5_	C_7_	C_8_	C_3′_	C_4′_	IC_50_ (µM)/Cell Viability (%)	Reference
PC3	H	OMe	OMe	OMe	H	OMe	17.2	[69]
	H	OH	OMe	OMe	H	OMe	11.8	
	H	OMe	OMe	OMe	OMe	OMe	80.0	[70]
VCaP	H	OMe	OMe	OMe	OMe	OMe	>80% (120 µM)	
	H	OH	OH	H	H	OH	>50% (50 µM)	[74]
DU145	OMe	OH	OMe	H	OH	OMe	<80% (50 µM)	[75]
	OMe	OH	OMe	OMe	H	OH	235.0	[76]
LNCaP	OMe	OH	OMe	OMe	H	OH	116.5	

## Data Availability

All data related to this research are presented in the manuscript.

## Abbreviations

SAR: structure–activity relationship; HBA: hydrogen bond acceptor; HBD: hydrogen bond donor; OCH_3_: methoxy group; OH: hydroxy group; IHB: intramolecular hydrogen bond; MF: methoxyflavone; DMF: dimethoxyflavone; TMF: trimethoxyflavone; TeMF: tetramethoxyflavone; PeMF: pentamethoxyflavone; HeMF: hexamethoxyflavone; Her-2+: epidermal growth factor receptor 2; AML: acute myeloid leukaemia; CML: chronic myeloid leukemia; logP: lipid partition coefficient; logD; distribution coefficient; TNFR: tumor necrosis factor receptor; MCL-1: Myeloid cell leukemia-1; BID: BH3 interacting-domain death agonist; BAX: Bcl-2-associated X protein; APAF-1: Apoptotic protease activating factor-1; CDK: cyclin-dependent kinase; MAPK: mitogen-activated protein kinase; GDP: guanosine diphosphate; GTP: guanosine triphosphatase; RAF: rapidly accelerated fibrosarcoma; MEK: mitogen-activated protein kinase; ERK1/2: extracellular signal-regulated kinase 1/2; GRB2: Growth Factor Receptor-bound protein 2; PI3K: phosphatidylinositol 3-kinase; AKT: protein kinase B; mTOR: mammalian target of rapamycin; NF-κB: nuclear factor kappa B: kappaB kinase; LPS: lipopolysaccharide; TNF-α: tumor necrosis factor alpha; PARP-1: Poly (ADP-ribose) polymerase 1; CAF: cancer-associated fibroblast; αSMA: alpha smooth muscle actin; MMP: matrix metalloproteinase; FAK: focal-adhesion kinase; JAK2: janus kinase 2; STAT3: signal transducer and activator of transcription 3; ACTN1: alpha-actinin-1; LC3: microtubule-associated protein light chain 3; PPARγ: peroxisome proliferator-activated receptor-gamma; ER: endoplastic reticulum; PERK: protein kinase R-like endoplasmic reticulum kinase; EIF2α: eukaryotic translation initiation factor 2 alpha; IRE1α: inositol-requiring enzyme 1 alpha; ATF4: activating transcription factor 4; CHOP: C/EBP homologous protein.
